# Genome-Wide Association Analysis Reveals the Genetic Basis of Iron-Deficiency Stress Tolerance in Maize

**DOI:** 10.3389/fpls.2022.878809

**Published:** 2022-06-02

**Authors:** Jianqin Xu, Weiya Xu, Xulei Chen, Huaqing Zhu, Xiuyi Fu, Futong Yu

**Affiliations:** ^1^Key Laboratory of Plant-Soil Interaction (MOE), Centre for Resources, Environment and Food Security, College of Resources and Environmental Sciences, China Agricultural University, Beijing, China; ^2^State Key Laboratory for Agrobiotechnology, Key Laboratory of Crop Heterosis and Utilization (MOE), Beijing Key Laboratory of Crop Genetic Improvement, China Agricultural University, Beijing, China; ^3^Key Laboratory of Maize DNA Fingerprinting and Molecular Breeding, Maize Research Center, Beijing Academy of Agriculture and Forestry Science (BAAFS), Beijing, China

**Keywords:** maize (*Zea mays* L.), iron (Fe) deficiency tolerance, genome-wide association study (GWAS), iron (Fe) acquisition strategies, candidate genes

## Abstract

Iron (Fe) is an essential trace element for almost all organisms and is often the major limiting nutrient for normal growth. Fe deficiency is a worldwide agricultural problem, which affects crop productivity and product quality. Understanding the Fe-deficiency response in plants is necessary for improving both plant health and the human diet. In this study, Fe-efficient (Ye478) and Fe-inefficient maize inbred lines (Wu312) were used to identify the genotypic difference in response to low Fe stress during different developmental stages and to further determine the optimal Fe-deficient Fe(II) supply level which leads to the largest phenotypic difference between Ye478 and Wu312. Then, genome-wide association analysis was performed to further identify candidate genes associated with the molecular mechanisms under different Fe nutritional statuses. Three candidate genes involved in Fe homeostasis of strategy II plants (strategy II genes) were identified, including *ZmDMAS1, ZmNAAT1*, and *ZmYSL11*. Furthermore, candidate genes *ZmNAAT1, ZmDMAS1*, and *ZmYSL11* were induced in Fe-deficient roots and shoots, and the expression of *ZmNAAT1* and *ZmDMAS1* responded to Fe deficiency more in shoots than in roots. Beyond that, several genes that may participate in Fe homeostasis of strategy I plants (strategy I genes) were identified, which were either encoding Fe transporters (*ZmIRT1* and *ZmZIP4*), or acting as essential ethylene signal transducers (*ZmEBF1*). Interestingly, *ZmIRT1, ZmZIP4*, and *ZmEBF1* were significantly upregulated under low Fe stress, suggesting that these genes may be involved in Fe-deficiency tolerance in maize which is considered as strategy II plant. This study demonstrates the use of natural variation in the association population to identify important genes associated with Fe-deficiency tolerance and may further provide insights for understanding the molecular mechanism underlying the tolerance to Fe-deficiency stress in maize.

## Introduction

Maize is one of the most widely grown crops in the world (Yang and Yan, 2021). The total maize production in 2018 equaled 1.12 billion tons (Jiang et al., 2020). Maize is also one of the most important crops that provide resources for animal feed and biofuel (Truelock et al., 2020). In addition, maize serves as the model system to study various biological phenomena, such as transposons, heterosis, and genetic diversity (Zhang et al., 2021). The annual planting area of maize is over 20 million hectares in China (He et al., 2020). Iron (Fe) is a key micronutrient of the various biological actions in plant systems, including photosynthesis, mitochondrial respiration, electron transfer reactions, and a yield-limiting factor for a variety of field crops around the world (Prerna et al., 2021; Sun et al., 2021). In agricultural production, there is an acute contradiction between Fe abundance in soils and Fe deficiency in crops (Li et al., 2014). Due to the tendency to form oxidized and hydroxide states under oxidizing, neutral, or alkaline soil conditions, plant-available ionic Fe concentrations in soil are very low, especially in calcareous and alkaline soils, which cover one-third of the world's cultivated areas (Rodríguez-Celma et al., 2013a). Fe deficiency results in decreased photosynthesis process that in turn causes reduction in the production of photosynthates and crop yields with total harvest loss (Prerna et al., 2021). Fe deficiency considerably restricts maize production, hence threatening food security (Long et al., 2020). Fe deficiency not only negatively affects the growth and development of plants, but also causes a high mortality rate, problems in pregnancy, low mental development, and physical health in humans (Murgia et al., 2012; Majeed et al., 2020). Fe deficiency is considered as the most common nutritional deficiency, which affects about two billion people (Sperotto et al., 2012; Bjørklund et al., 2017). About 30% of the world's population has anemia, and about 50% of these cases are due to Fe deficiency (Kassebaum et al., 2014; Bjørklund et al., 2017).

Plants have evolved two distinct strategies to solubilize and transport Fe to acclimate Fe-deficiency stress conditions: the reduction strategy of non-graminaceous plants and the chelation strategy of graminaceous plants (Kobayashi et al., 2014). Strategy I and strategy II are thought to be mutually exclusive (Römheld and Marschner, 1986). However, recent studies uncovered that the distinction between strategy I and strategy II is becoming blurry, questioning the validity of the concept of mutual exclusivity (Grillet and Schmidt, 2019). For example, secretion of iron-mobilizing compounds, such as coumarins by roots of strategy I species (Tsai and Schmidt, 2017), and the strategy I plants can also benefit from the PS system conceptually confined to grasses (Suzuki et al., 2016). Among them, in non-grass species, the coumarins have emerged as key players for Fe solubilization and/or uptake, in particular when the pH of the soil is high and the FRO2 activity is reduced (Tsai and Schmidt, 2017). For example, sideretin (a redox-active catecholic metabolite) is the major coumarin exuded into the rhizosphere in response to iron deficiency under acidic conditions, whereas alkaline conditions stimulate the exudation of less-oxidized coumarins, such as fraxetin (Rajniak et al., 2018). Both sideretin and fraxetin efficiently mobilize and reduce insoluble Fe(III), and rescue chlorotic phenotypes of susceptible mutants and wild-type plants grown under conditions of low iron availability (Rajniak et al., 2018). Moreover, the co-expression network constructed from iron-responsive genes in rice transcriptomes reveals a remarkably similar architecture to the networks inferred for Arabidopsis (Schmidt and Buckhout, 2011; Rodríguez-Celma et al., 2013b), indicating that the iron regulon is, in large parts, evolutionarily conserved in strategy I and strategy II plants (Grillet and Schmidt, 2019).

Gene regulatory network on Fe homeostasis is an important evolutionary product in plants for coping with fluctuating environments. The key regulator in nongraminaceous plants was first identified, encoding a basic helix–loop–helix (bHLH) transcriptional regulator, *T3238FER* (*FIT*), which responds to Fe deficiency and interacts with *AtbHLH38/39/100/101* (forming heterodimers) to regulate Fe utilization (Kobayashi and Nishizawa, 2012; Li X. et al., 2016). Under Fe deficiency, FIT was shown to be essential for positively regulating the high-level induction of the *IRT1, AHA2*, and *FRO2* genes (Ivanov et al., 2012), some of which were proposed to be its direct targets (Wang et al., 2012). *FRO2* is transcriptionally regulated by FIT, while *IRT1* is both transcriptionally and post-transcriptionally regulated by FIT (Colangelo and Guerinot, 2004).

Another important regulatory network involved in Fe-deficiency responses is the POPEYE (PYE) network. PYE distinguishes from FIT-mediated positive regulation and functions as a repressor in the Fe-deficiency regulatory network, as well as its homolog IRO3 in rice (Long et al., 2010; Zheng et al., 2010). Two crucial genes from this regulatory system are the bHLH transcription factor (*bHLH047*), named *PYE*, and the putative E3-ubiquitin ligase, called *BRUTUS* (*BTS*) (Hindt and Guerinot, 2012). Among them, PYE appears to directly repress the induction of Fe-deficiency-related genes (including *NAS4, FRO3*, and *ZIF1*) and optimizes Fe redistribution and root growth under Fe deprivation conditions (Kobayashi et al., 2014; Wang et al., 2018). It is localized to the nuclei of all cells within Fe-deficient roots. BTS, also possessing a hemerythrin domain for Fe binding, may negatively regulate Fe homeostasis-related genes (Long et al., 2010). Two other members of the PYE regulatory network, MYB10 and MYB72, which are members of the myeloblastosis (MYB) family of transcription factors, have been implicated in Fe redistribution through the regulation of *NAS4* (Palmer et al., 2013). In addition, MdMYB58 modulates Fe homeostasis by directly binding to the *MdMATE43* promoter in apple (*Malus domestica*) (Wang et al., 2018).

The *cis*-acting elements IDE1 (Fe-deficiency-responsive element 1) and IDE2 were first found in graminaceous crops induced by Fe-deficiency stress (Kobayashi et al., 2003). They confer Fe-deficiency-inducible expression in tobacco roots, as well as in rice roots and leaves (Ogo et al., 2007). OsIDEF1 and 2 (IDE binding Factor), belonging to the ABI3/VP1 and NAC plant-specific transcription factor families, respectively, have also been identified as positive regulators of the Fe-deficiency response (Kobayashi et al., 2007). OsIDEF1 and 2 bind specifically to IDE1 and 2, specific sequences: CATGC and CA(A/C)G(T/C)(T/C/A) (T/C/A), respectively (Kobayashi et al., 2014). Both of them function as key components regulating the response to and tolerance of Fe deficiency (Ogo et al., 2008). The Fe-deficiency-inducible bHLH transcription factor, OsIRO2, is strongly induced under Fe deficiency and is positively regulated by IDEF1 (Kobayashi et al., 2009). The core sequence for OsIRO2 binding (CACGTGG) is often present among Fe-deficiency-inducible gene promoters in rice (Ogo et al., 2006). Microarray analysis demonstrated that OsIRO2 regulates 59 Fe-deficiency-induced genes (Ogo et al., 2007), including *OsNAAT1, OsDMAS1, OsNAS1, OsNAS2, TOM1*, and *OsYSL15* (Ogo et al., 2011).

In addition, Fe-deficiency stress is signaled by many plant hormones, including ethylene, cytokinins, gibberellins, auxins, brassinosteroids (Ivanov et al., 2012; Wang et al., 2012; Zamboni et al., 2012). Yeast two-hybrid screening revealed that FIT also interacts with the ETHYLENE INSENSITIVE 3 (EIN3) and ETHYLENE INSENSITIVE 3-LIKE1 (EIL1) transcription factors (Lingam et al., 2011). These interactions, which promote Fe uptake, are required for full FIT accumulation and contribute to full FIT downstream target gene expression. Moreover, the DELLA gibberellin signaling repressors competitively interact with FIT to inhibit its transcriptional activity in root epidermal cells (Wild et al., 2016).

To cope with a low Fe environment, plants have evolved elaborate mechanisms underlying Fe homeostasis *via* intricate transcriptional and post-transcriptional regulation (Wang et al., 2018). But so far, there is relatively little information about the complex Fe homeostasis system. This is mainly because the identification of related genes is still limited. So far, the use of GWAS has mainly focused on the genetic information related to seed Fe concentration in a variety of crops, such as chickpea (Upadhyaya et al., 2016), wheat (Alomari et al., 2018; Arora et al., 2019; Cu et al., 2020; Wang et al., 2021), barley (Mamo et al., 2014), millet (Jaiswal et al., 2018; Pujar et al., 2020), rice (Zhang et al., 2018; Bollinedi et al., 2020), and maize (Benke et al., 2015; Hindu et al., 2018). There are also works on the genetic basis of soybean Fe-deficiency chlorosis using GWAS (Mamidi et al., 2014; Assefa et al., 2020). However, there is limited information on the identification of genes related to low Fe stress in maize using GWAS methods. To shed light on the metabolic pathway of Fe and to identify the genes and transcription factors that are responsible for the uptake of Fe in plants are crucial for plant growth, development, and improvement of biofortified crops (Li et al., 2014). Therefore, this study used GWAS in maize to identify important genes associated with Fe homeostasis under low Fe stress conditions. Moreover, in paddy fields, Fe^2+^ is abundant because of the low pH and low oxygen availability, which is assumed to be the reason why rice plants possess Fe^2+^ uptake systems (Masuda et al., 2017). Under hydroponic conditions, this study used 2,2′-bipyridine to supply Fe^2+^ to identify genes that respond to Fe starvation in maize. This can analyze whether a rice-like Fe-utilizing mechanism exists in maize with broad genetic diversity.

## Materials and Methods

### Experimental Design

#### Experiments 1 and 2: Variations in Tolerance to Fe Deficiency Between Fe-Efficient and Fe-Inefficient Inbred Lines

**Figure 1:**
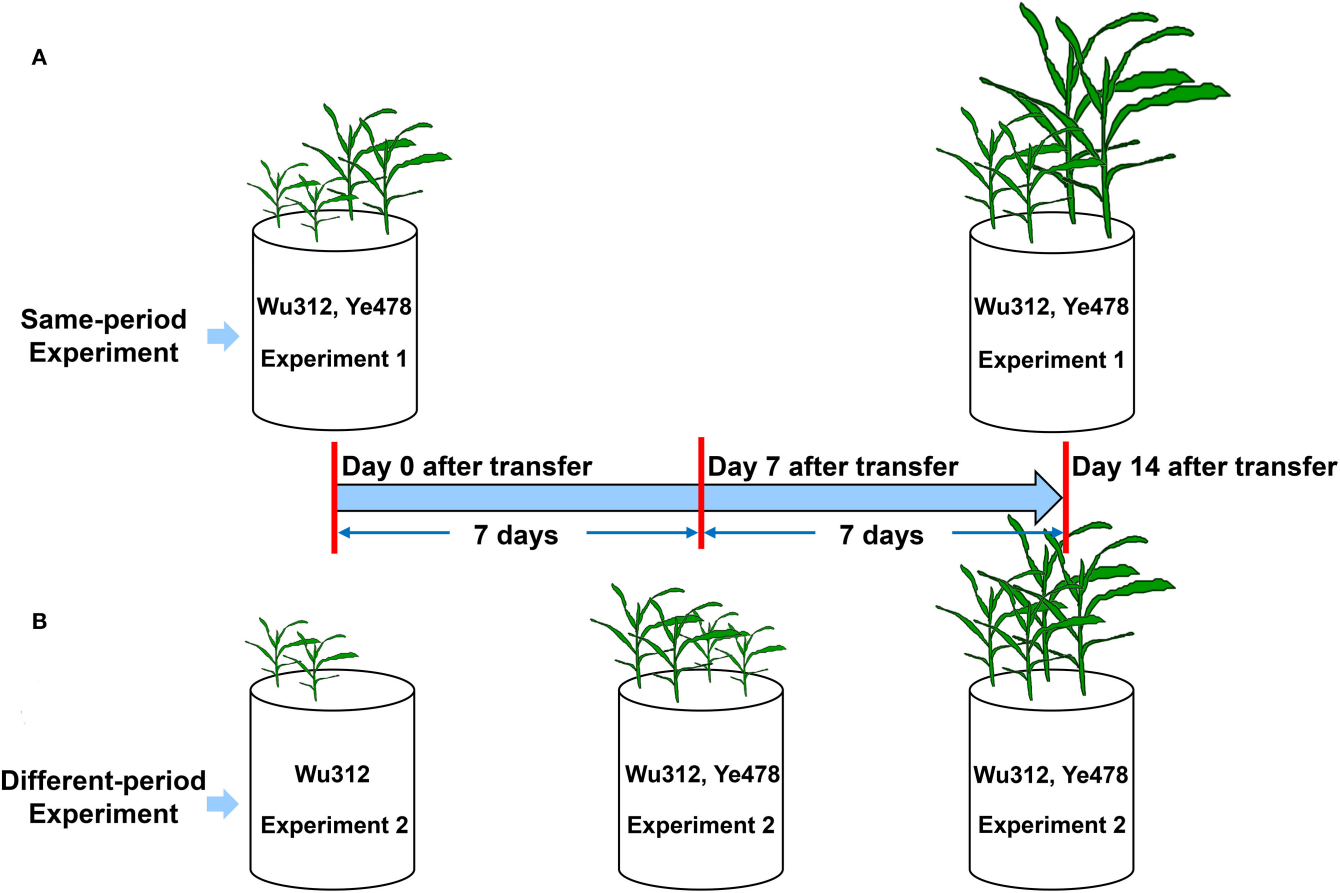
Experimental setup in Experiments 1 and 2. **(A)** In Experiment 1, two seedlings of Fe-inefficient inbred line Wu312 and Fe-efficient inbred line Ye478 were simultaneously grown in hydroponics for 14 days. **(B)** In Experiment 2, only two seedlings of Wu312 were transferred at the same time with Experiment 1, and then, two seedlings of Ye478 were transferred 7 days later.

In Experiment 1, two seedlings of Fe-inefficient inbred line Wu312 and Fe-efficient inbred line Ye478 were simultaneously grown in hydroponics for 14 days (Figure 1A). In Experiment 2, only two seedlings of Wu312 were transferred at the same time with Experiment 1, and then, two seedlings of Ye478 were transferred 7 days later (Figure 1B). Ten different Fe(II)-2,2′-bipyridyl levels (0.03, 0.06, 0.09, 0.3, 0.6, 0.9, 1.6, 2.5, 5, and 10 μmol L^−1^) were designed to determine the optimal Fe-deficient supply level.

#### Experiment 3: Genome-Wide Association Study (GWAS) for Fe-Deficiency Tolerance in Maize

The association panel AM508 was used to analyze the association between markers and the traits associated with Fe-deficiency tolerance. This panel consisted of 1.03 million high-quality SNPs genotyped by RNA-seq and 56,110 SNPs genotyped by the MaizeSNP50 BeadChip (Li et al., 2013). A total of 559,285 high-quality SNPs with minor allele frequency (MAF) above 0.05 were used in the present research. Experiments were performed under Fe-deficient [0.6 μmol L^−1^ Fe(II)-2,2′-bipyridyl] and Fe-sufficient conditions [350 μmol L^−1^ Fe(II)-EDTA].

#### Experiment 4: Expression of Candidate Genes

Seedlings of Ye478 were hydroponically grown under Fe-deficient [-Fe: 0.6 μmol L^−1^ Fe(II)-2,2′-bipyridyl] and Fe-sufficient [CK: 350 μmol L^−1^ Fe(II)-EDTA] conditions for 14 days. The expression of candidate genes was analyzed in the shoots and roots of Ye478, including *ZmNAAT1* (GRMZM2G096958), *ZmDMAS1* (GRMZM2G060952), *ZmYSL11* (GRMZM5G812538), *ZmIRT1* (GRMZM2G118821), *ZmZIP4* (GRMZM2G111300), and *ZmEBF1* (GRMZM2G171616). Each treatment contained three biological replications. Three technical replications were performed for each biological replication.

### Plant Growth

Maize seeds were sterilized for 30 min in a 10% solution of H_2_O_2_, washed with distilled water, soaked in saturated CaSO_4_ for 10 h, and then germinated on moist filter paper in the dark at room temperature. Two days later, the germinated seeds were wrapped in moist filter paper roll and grown. At the stage of two visible leaves, the seedlings were selected and transferred into a 40-L black container. Solution pH was set at 5.5–6.0. The adjusted Hoagland nutrient solution contained the following parameters (mmol L^−1^): 0.5 NH_4_NO_3_, 0.5 CaCl_2_, 1.5 Ca(NO_3_)_2_, 0.75 K_2_SO_4_, 0.65 MgSO_4_, 0.1 KCl, 0.25 KH_2_PO_4_, 1.0 × 10^−3^ H_3_BO_3_, 0.01 Zn-EDTA, 8.0 × 10^−3^ CuSO_4_, 1.2 × 10^−2^ MnSO_4_, 4.0 × 10^−5^ (NH_4_)Mo_7_O_24_, and 4.0 × 10^−3^ NiCl. All the experiments were conducted in randomized blocks with three replications. All the experiments were conducted in the growth chamber under strictly controlled environments. The condition of the growth chamber was set as a 14-h light period from 8:00 to 22:00 with 28 °C and a 10-h dark period with 22 °C. The average light intensity measured at the canopy was 350 μmol m^−2^ s^−1^.

### Data Collection

Experiments were terminated 14 days after transplanting. During the period from 9:00 to 11:00 A.M., SPAD values of the youngest fully expanded leaf were measured on the 1/3 part from the leaf base for three times using the SPAD-502 leaf chlorophyll meter. In addition, the mean of three observed values was recorded for each plant. The plant height and root length for each plant were measured. All samples were heat-treated at 105°C for 30 min and dried at 75°C till constant weight. Concentrations of Fe, copper (Cu), and zinc (Zn) in shoots and roots were analyzed by ICP-AES (inductively coupled plasma-atomic emission spectroscopy). Relative ratios of root to shoot (R/S) efficiency and nutrient contents were estimated using the following Equations (1) and (2), respectively.

$$\begin{array}{l} \left(1\right)\text{ Relative ratios of R}/\text{S }=\text{ R}/\text{S }\left(-\text{Fe}\right)\;/\text{ R}/\text{S }\left(\text{CK}\right)\\ \left(2\right)\text{ Nutrient content }=\text{ Concentration }\times \text{ Dry weight} \end{array}$$

For GWAS, Fe score for each plant has been visually recorded for three times since the 12th day after transplanting. Five scales (0–4) were designed to assess the ability to tolerate Fe deficiency of each line in the GWAS panel under Fe-deficient condition [-Fe: 0.6 μmol L^−1^ Fe(II)-2,2′-bipyridyl] (Supplementary Figure S1). Under Fe-deficient condition, Fe-deficiency tolerance scoring mainly depends on the degree of inhibition in growth and development for the whole plant, as well as the degree of chlorosis of young leaves. Score 0: plants developed about three leaves with one sprout and showed the severest stunted growth and reduced plant height, distorted leaf growth, and yellowish-white necrotic lesions distributed on the whole youngest leaves. Score 1: plants developed four leaves and one sprout and showed reduced plant height and wrinkled leaf margins. Fe-deficient chlorosis with necrotic patches was distributed on the youngest leaves. Score 2: plants developed four leaves and one sprout and exhibited a better growth compared with Score-0 and Score-1 plants, but still showed chlorosis on the youngest leaves. Score 3: plants developed four leaves and one sprout and obtained an improved plant height compared with Score-2 plants. Young leaves showed Fe-deficient chlorosis without wrinkled leaf margins. Score 4: plants grew well without reductions in plant height but still display banded chlorosis in the middle of young leaves.

### RNA Extraction and Gene Expression Quantification

In Experiment 4, total RNA was extracted from shoots and roots of plants using the Total RNA Extraction Kit (TIANGEN, China). The cDNA was synthesized in accordance with Fast Quant RT Super Mix Reverse Transcription Kit instructions (Transgene, Beijing, China). Quantitative real-time PCR was performed using SYBR Green Real-time RT-PCR (Takara) and an ABI7500 Fast Real-Time PCR System (Applied Biosystems). The relative gene expression level was calculated using the 2^−ΔΔCt^ method. Each real-time PCR experiment contained three technical replicates.

### Statistical Analysis

The means for each trait were compared using one-way ANOVA at a 0.05 level of probability followed by the least significant difference (LSD) test using SPSS 20.0. The linear mixed effect function lmer in the lme4 package of R version 3.1.1 was fitted to each RIL to obtain the BLUP (best linear unbiased prediction) value for each trait.

### Genome-Wide Association Analysis

A genome-wide association analysis on the traits associated with Fe-deficiency tolerance was performed using a mixed linear model (MLM) (Li et al., 2013) which considered the population structure and relative kinship matrix (Yu et al., 2006) using TASSEL 3.0. Using “no compression” and “population parameters previously determined” (P3D) algorithms, the MLM program in TASSEL 3.0 was conducted to detect the association between the phenotype and genotype. The threshold of *P* = 1 × 10^−4^ was used to determine a significant SNP, which was determined based on the quantile–quantile plots and distribution of *P*-values for all the traits (Supplementary Figure S2).

### Identification and Annotation of Candidate Genes

According to the previous study, the LD distance of this association panel was 50 kb (Li et al., 2013). Therefore, candidate genes within a 100-kb region (50 kb upstream and downstream of the lead SNP) flanking each leading SNP were identified. Their functional descriptions were identified using the maize B73 reference genome assembly version 2 available on the MaizeGDB Database (http://www.maizeGDB.org/) and Gramene Database (https://www.gramene.org/).

## Results

### Difference in Fe-Deficiency Tolerance Between Fe-Efficient and Fe-Inefficient Maize Inbred Lines

**Figure 2:**
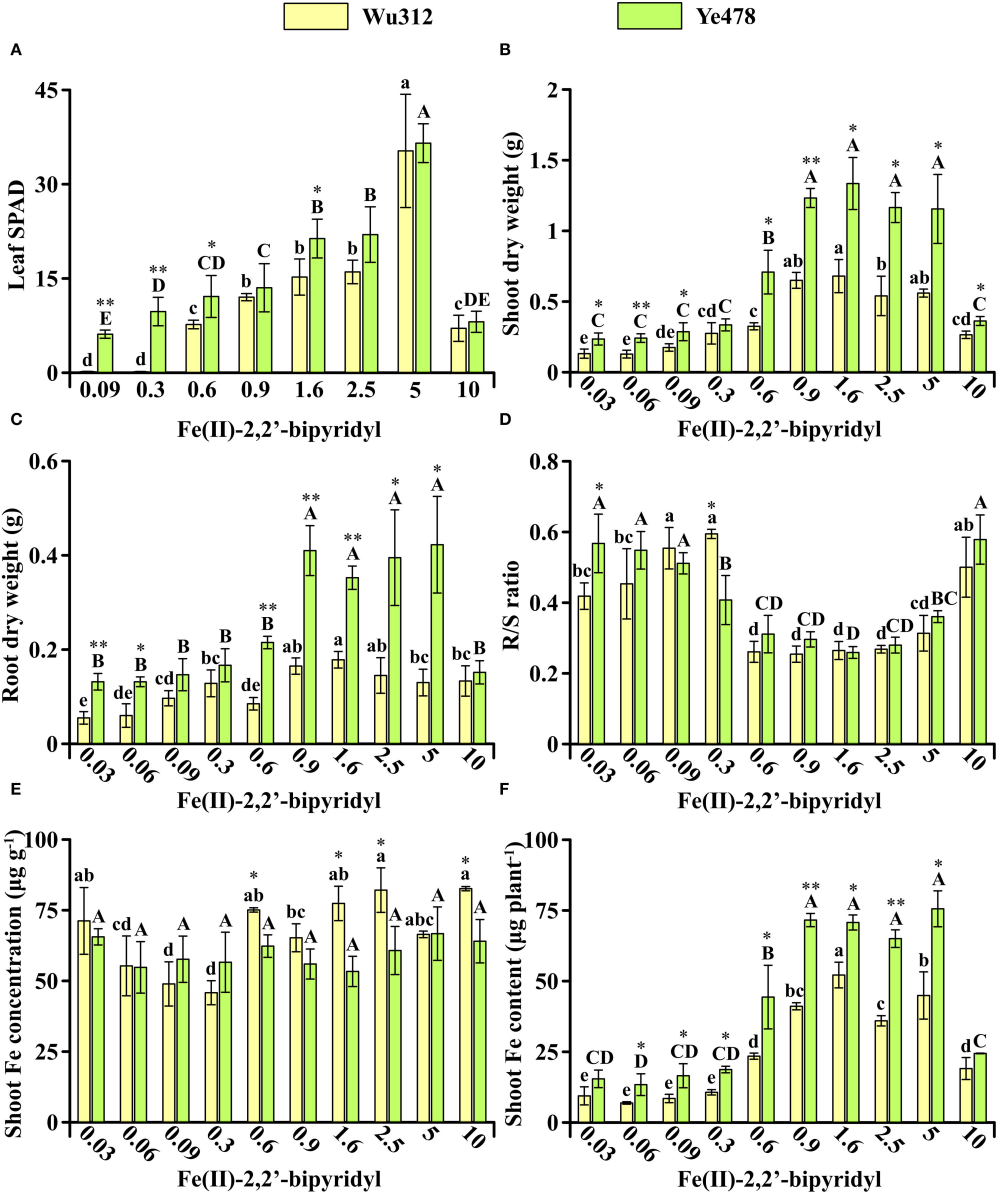
Effects of different Fe supply levels on plant growth of Wu312 and Ye478 during the same period in Experiment 1. **(A)** Leaf SPAD; **(B)** shoot dry weight; **(C)** root dry weight; **(D)** R/S ratio; **(E)** shoot Fe concentration; **(F)** shoot Fe content. Different lowercase (uppercase) letters indicate significant differences in Wu312 (Ye478) among different treatments at *P* < 0.05. * and ** indicate significant differences between Wu312 and Ye478 in the same treatment at *P* < 0.05 and *P* < 0.01, respectively.

In Experiment 1, Fe-efficient inbred line Ye478 and Fe-inefficient inbred line Wu312 were hydroponically grown during the same period. Compared with Wu312, Ye478 exhibited significantly higher leaf SPAD, shoot and root dry weight (Figure 2). On the Fe-deficient supply of 0.6 μM Fe(II)-2,2′-bipyridyl, the leaf SPAD, shoot, and root dry weight, and shoot Fe content of Ye478 were 1.4-fold, 2.2-fold, 2.5-fold, and 1.2-fold higher than those of Wu312, respectively (Figures 2A–C,F). Especially, the largest difference in shoot dry weight between two inbred lines was found at the ferrous concentration of 0.6 μM among ten different treatments (Figure 2B).

We hypothesized that compared to Wu312, the roots of Ye478 had the genetic advantage when seedlings were transplanted. When the two inbred lines were hydroponic cultured together, Ye478 displayed an absolute advantage in competition under the condition of a limited total amount of Fe supply. To test the above hypothesis, we set up this experiment at different developmental stages.

**Figure 3:**
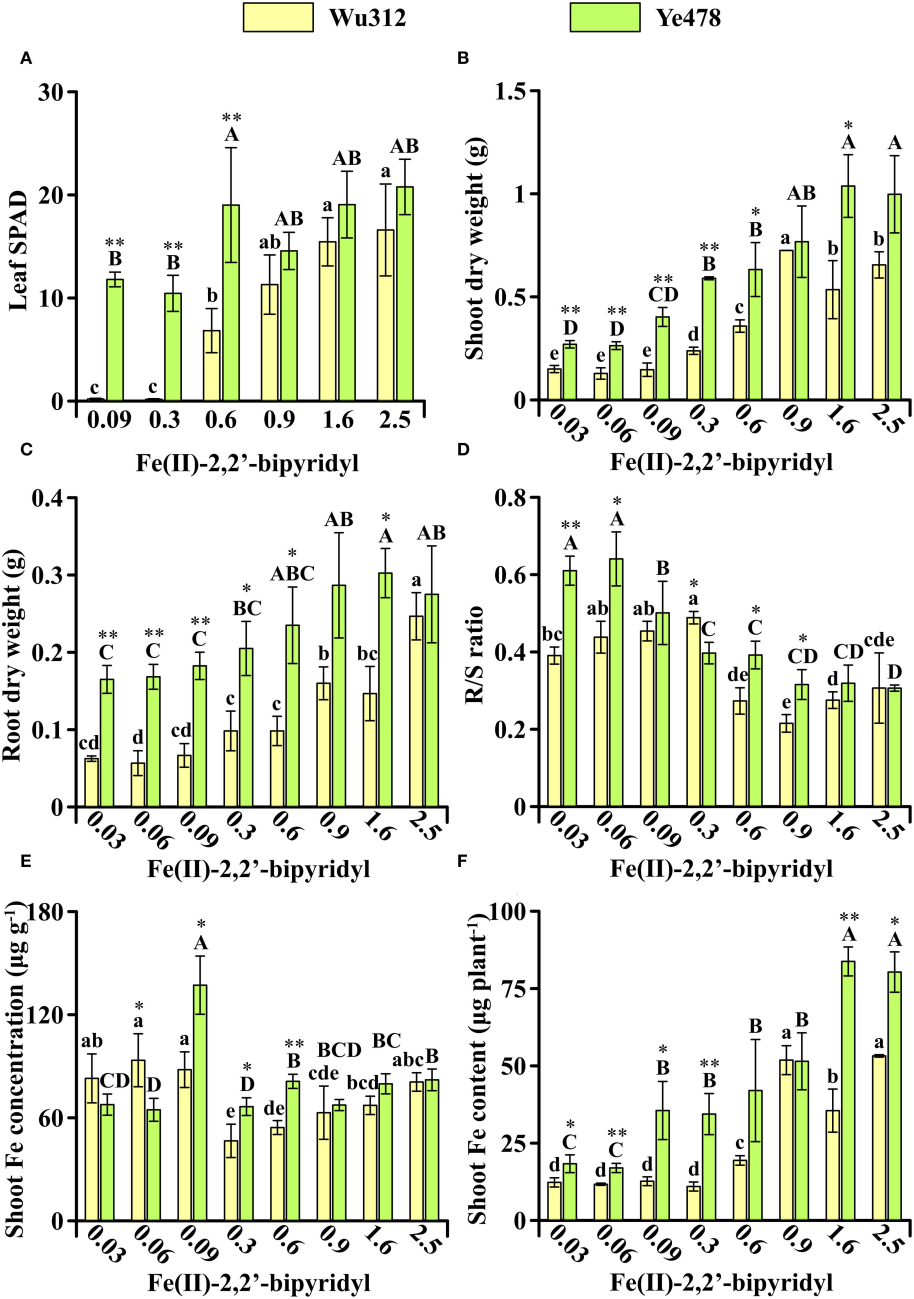
Effects of different Fe supply levels on plant growth of Wu312 and Ye478 during different periods in Experiment 2. **(A)** Leaf SPAD; **(B)** shoot dry weight; **(C)** root dry weight; **(D)** R/S ratio; **(E)** shoot Fe concentration; **(F)** shoot Fe content. Different lowercase (uppercase) letters indicate significant differences in Wu312 (Ye478) among different treatments at *P* < 0.05. * and ** indicate significant differences between Wu312 and Ye478 in the same treatment at *P* < 0.05 and *P* < 0.01, respectively.

In Experiment 2, compared with Wu312, Fe-efficient inbred line Ye478 was transplanted and grown in hydroponics 7 days later. Although Wu312 has been grown for 14 days after transplanting, the 7-day Ye478 still displayed advantages in plant growth under Fe deficiency (Figure 3). On the supply of 0.6 μM ferrous concentration, the leaf SPAD, shoot, and root dry weight, and shoot Fe content of 7-day Ye478 were 2.8-fold, 1.8-fold, 2.4-fold, and 2.2-fold higher than those of 14-day Wu312, respectively (Figures 3A–C,F).

Compared to the phenotypes at the same developmental stage in Experiment 1, plant growth of Wu312 should be significantly improved when grown 7 days earlier in Experiment 2. However, the results of this experiment refuted our original hypothesis. Because even when the roots of Wu312 had a significant advantage over Ye478, Wu312 eventually developed to the fourth leaf and then stopped growing in Experiment 2, and the phenotypes of Wu312 in Experiment 2 were similar to the phenotypes at the same development stage in Experiment 1.

The difference in the ability to tolerate Fe deficiency between the two different genotypes, which have been observed in Experiments 1 and 2, cannot be explained clearly by physiological traits. Therefore, the GWAS panel which has extensive genetic variation was utilized to perform genome-wide association analysis and further identify candidate genes responding to low Fe stress, preliminarily revealing the genetic basis conferring Fe-deficiency tolerance in maize.

### Genome-Wide Association Analysis of Fe-Deficiency Tolerance

**Figure 4:**
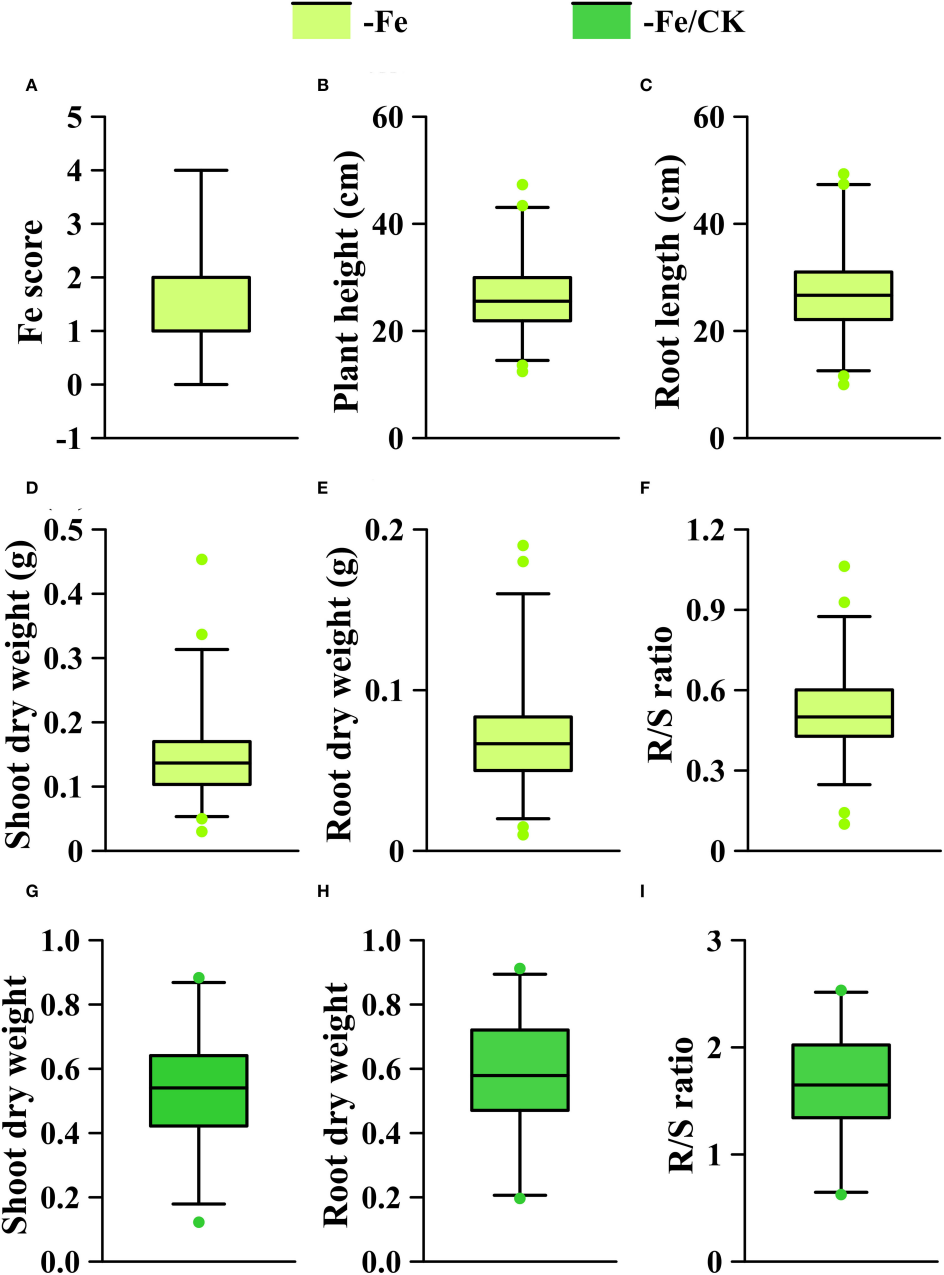
Phenotypic distribution of each trait under different conditions (-Fe, -Fe/CK) in the GWAS panel. **(A)** Fe score; **(B)** plant height; **(C)** root length; **(D,G)** shoot dry weight; **(E,H)** root dry weight; **(F,I)** R/S ratio. The solid line in each box represents the median values. The upper and bottom lines represent the 99th and 1st percentiles, respectively, and the top and bottom edges of each box represent the 75 th and 25 th percentiles, respectively. The solid dots represent outliers.

The phenotypic means of each trait in the GWAS panel are shown in Supplementary Table S1. On average, Fe deficiency decreased the means of plant height, root length, shoot dry weight, and root dry weight by 38.6, 30.6, 51.7, and 46.2%, respectively, and the mean of R/S ratio was increased by 75.9% under the Fe-deficient condition. To identify the loci associated with Fe efficiency, we used all traits in the -Fe treatment, shoot and root dry weights, and R/S ratio in the -Fe/CK treatment to perform GWAS. All the traits of the GWAS panel varied widely in the -Fe or the -Fe/CK treatments (Figure 4). The coefficients of variance for each trait under Fe deficiency ranged from 22.8 to 71.6% (Supplementary Table S1). Furthermore, significant correlations were observed among plant height, root length, shoot, and root dry weights under Fe deficiency (Supplementary Table S2).

**Figure 5:**
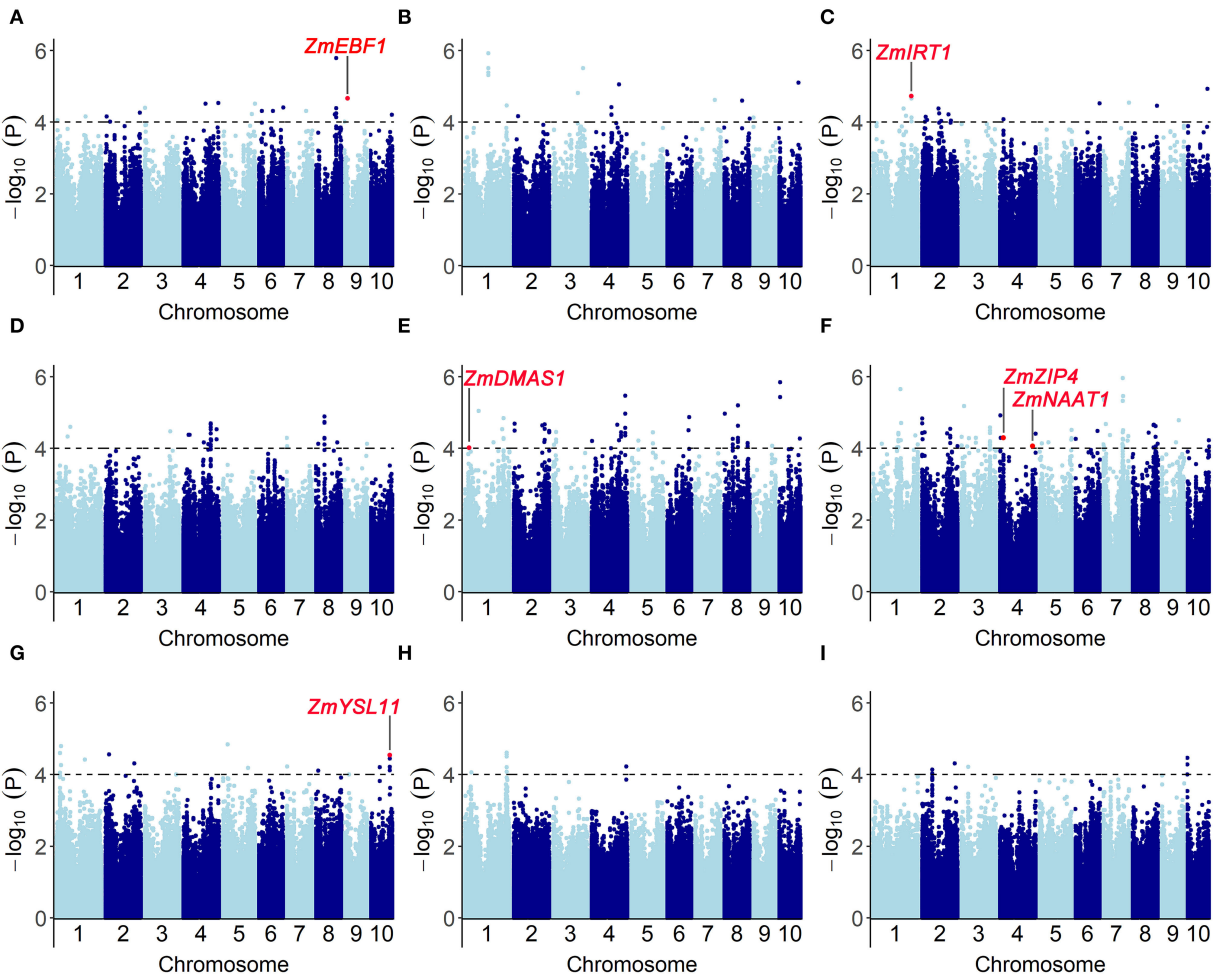
Genome-wide association results for the traits associated with Fe-deficiency tolerance in a maize association population under different conditions (-Fe, -Fe/CK). Manhattan plots for **(A)** Fe score (FeSc), **(B)** plant height (PH), **(C)** root length (RL), [-Fe: **(D)**; -Fe/CK: **(G)**] shoot dry weight (SDW), [-Fe: **(E)**; -Fe/CK: **(H)**] root dry weight (RDW), [-Fe: **(F)**; -Fe/CK: **(I)**] R/S are shown. A total of 353 significant SNPs were identified at a significant level of *P* < 1 × 10^−4^. Gray dashed lines correspond to the thresholds of *P* < 1 × 10^−4^. Red dots refer to the significant SNPs that contain the genes associated with Fe-deficiency tolerance, including *ZmEBF1* (GRMZM2G171616), *ZmIRT1* (GRMZM2G118821), *ZmDMAS1* (GRMZM2G060952), *ZmZIP4* (GRMZM2G111300), *ZmNAAT1* (GRMZM2G096958), and *ZmYSL11* (GRMZM5G812538).

We performed a genome-wide association study by fitting a mixed linear model (MLM) with population structure and familial relatedness. The quantile–quantile plots (Q-Q plots) for GWAS based on BLUP value of each trait implied that the associations were well controlled for population structure (Supplementary Figure S2). At a significance level of *P* < 1 × 10^−4^, a total of 353 significant SNPs were mapped on 10 chromosomes (Figure 5, Supplementary Table S3): 16 SNPs for Fe score, 21 SNPs for plant height, 22 SNPs for root length, 63 SNPs for shoot dry weight, 85 SNPs for root dry weight, and 146 SNPs for R/S ratio. For all traits, the phenotypic variance explained by each SNP varied from 5.1 to 13.3%.

### Identification of Candidate Genes

Based on the physical position (B73_v2 reference), 472 candidate genes were identified within 100-kb flanking 353 significant SNPs which were associated with Fe score, plant height, root length, shoot and root dry weight, and R/S ratio (Supplementary Table S3). According to the functional descriptions of total 472 candidate genes in *Arabidopsis* and rice on MaizeGDB Database (http://www.maizeGDB.org) and Gramene Database (https://www.gramene.org), seven genes were proposed to be associated with Fe-deficiency tolerance in maize, including the genes participating in Fe homeostasis of strategy I plants and strategy II plants (Table 1).

**Table 1:** Candidate genes associated with Fe-deficiency tolerance identified by GWAS in maize.

**Chr**	**Trait**	**SNP**	**Gene ID**	**Position (bp)^a^**	**Annotation**
1	RDW	chr1.S_31743584	GRMZM2G060952	31744679-31747784	ZmDMAS1–Deoxymugineic acid synthase 1
1	RL	chr1.S_258444638	GRMZM2G118821	258353073-258355277	ZmIRT1–Iron-regulated transporter 1
4	R/S	chr4.S_26684019	GRMZM2G111300	26655455-26658141	ZmZIP4–Zinc-regulated, iron-regulated transporter-like protein 4
4	R/S	chr4.S_219582768	GRMZM2G096958	219581608-219585123	ZmNAAT1–Nicotianamine aminotransferase 1
9	FeSc	chr9.S_90366645	GRMZM2G171616	90365166-90368247	ZmEBF1–EIN3-binding F-Box protein 1
10	SDW	chr10.S_131843508	GRMZM5G812538	131884181-131884942	ZmYSL11–Yellow stripe-like transporter 11

a *Physical position accords B73_v2 reference*.

Among them, GRMZM2G060952, also known as *ZmDMAS1* which encodes deoxymugineic acid synthase, was located in the 1.1 kb downstream of the SNP chr1.S_31743584 which was significantly associated with root dry weight. GRMZM2G096958, also known as *ZmNAAT1* which encodes nicotianamine aminotransferase, was containing the SNP chr4.S_219582768 that is significantly correlated with R/S ratio under Fe deficiency. GRMZM5G812538 also known as *ZmYSL11* encoding a yellow stripe-like transporter, was identified within the overlapped regions co-localized by chr10.S_131843508 and *qFe(II)-SPAD10-2* which is a reported QTL controlling leaf SPAD detected in the Ye478 × Wu312 RIL population under Fe deficiency (Xu et al., 2022).

Apart from strategy II genes, two candidate genes probably associated with Fe homeostasis in strategy I plants were identified by two significant SNPs. GRMZM2G118821, encoding an iron transporter IRT in maize, was detected in the 89.4 kb upstream of the significant SNP chr1.S_258444638 which was associated with root length under Fe deficiency. GRMZM2G111300, which encodes another member of ZIP (ZRT/IRT-like protein) family, was localized in the 25.9 kb upstream of chr4.S_26684019 on chromosome 4. GRMZM2G171616, encoding an EIN3-binding F-Box protein that may be involved in the regulation of ethylene signaling and Fe homeostasis in strategy I plants, was identified within the co-localization of chr9.S_90366645 and *qFe(II)-SPAD9-1* which is another locus mapped on chromosome 9 in the Ye478 × Wu312 RIL population reported by Xu et al. (2022).

### Expression of Candidate Genes in Different Tissues

To evaluate the effect of Fe deficiency on the expression of candidates participating in Fe homeostasis of strategy II and strategy I plants, the relative expression levels of these genes were analyzed in the shoots and roots of Ye478 under Fe-deficient [-Fe: 0.6 μmol L^−1^ Fe(II)-2,2′-bipyridyl] and Fe-sufficient [CK: 350 μmol L^−1^ Fe(II)-EDTA] conditions.

**Figure 6:**
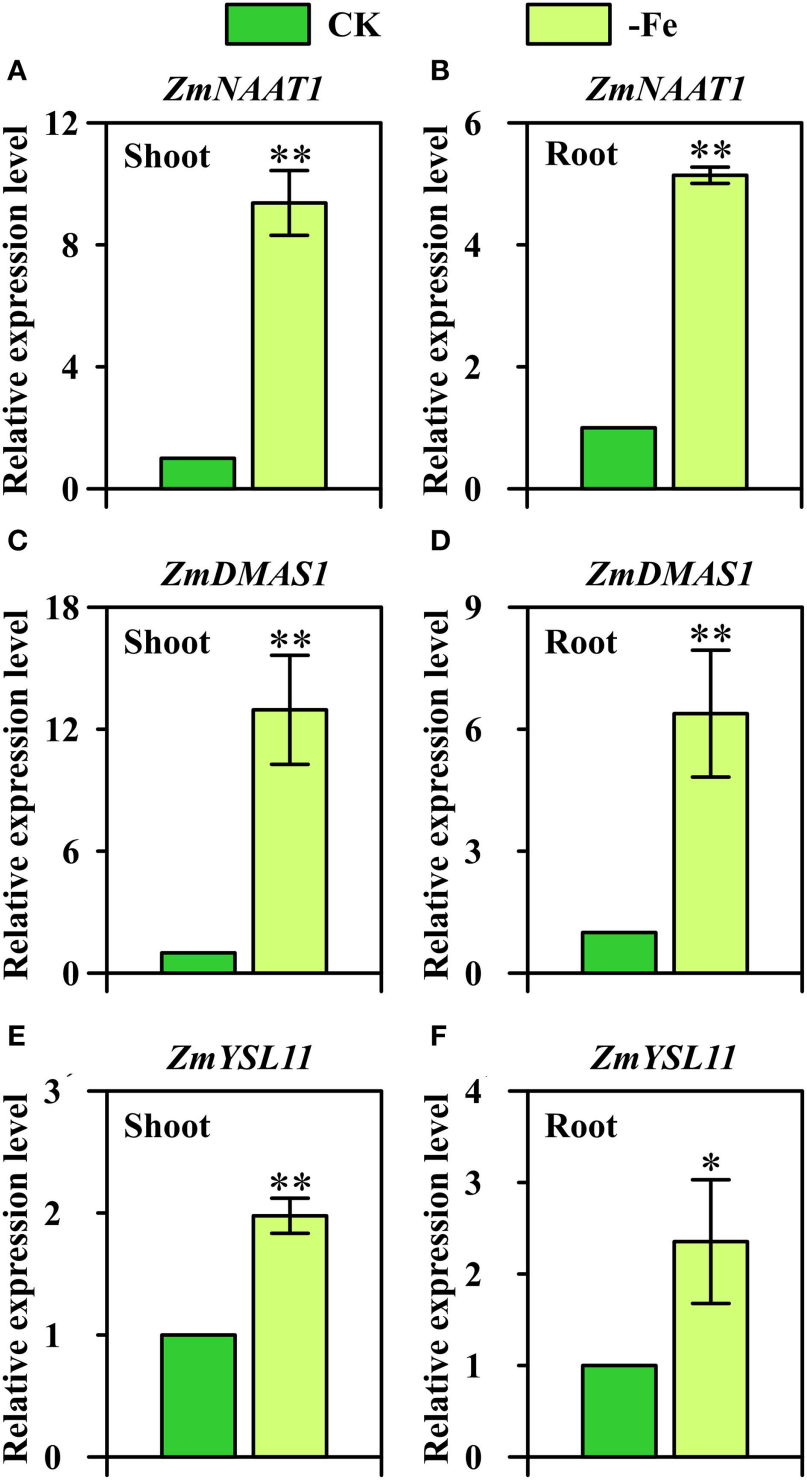
Relative expression levels of strategy II genes **(A,B)** *ZmNAAT1* (GRMZM2G096958), **(C,D)** *ZmDMAS1* (GRMZM2G060952), and **(E,F)** *ZmYSL11* (GRMZM5G812538) in the shoots **(A,C,E)** and roots **(B,D,F)** of Ye478 grown under Fe-deficient (-Fe) and Fe-sufficient (CK) conditions. * and ** indicate significant differences between treatments at *P* < 0.05 and *P* < 0.01, respectively.

Overall, strategy II genes *ZmNAAT1* (GRMZM2G096958), *ZmDMAS1* (GRMZM2G060952), and *ZmYSL11* (GRMZM5G812538) were all significantly upregulated in both shoots and roots under low Fe stress (Figure 6). In the shoots, 9.4-fold and 13.0-fold upregulation were observed in the expression of *ZmNAAT1* and *ZmDMAS1*, respectively (Figures 6A,C). By contrast, the relative expression levels of *ZmNAAT1* and *ZmDMAS1* in the roots were 5.1-fold and 6.4-fold upregulated under Fe deficiency, respectively. These results indicated that the expression of *ZmNAAT1* and *ZmDMAS1* responded to Fe deficiency more in shoots than in roots. For *ZmYSL11*, 2.0-fold and 2.4-fold upregulations were found in the shoots and roots under Fe-deficient conditions, respectively (Figures 6E,F).

**Figure 7:**
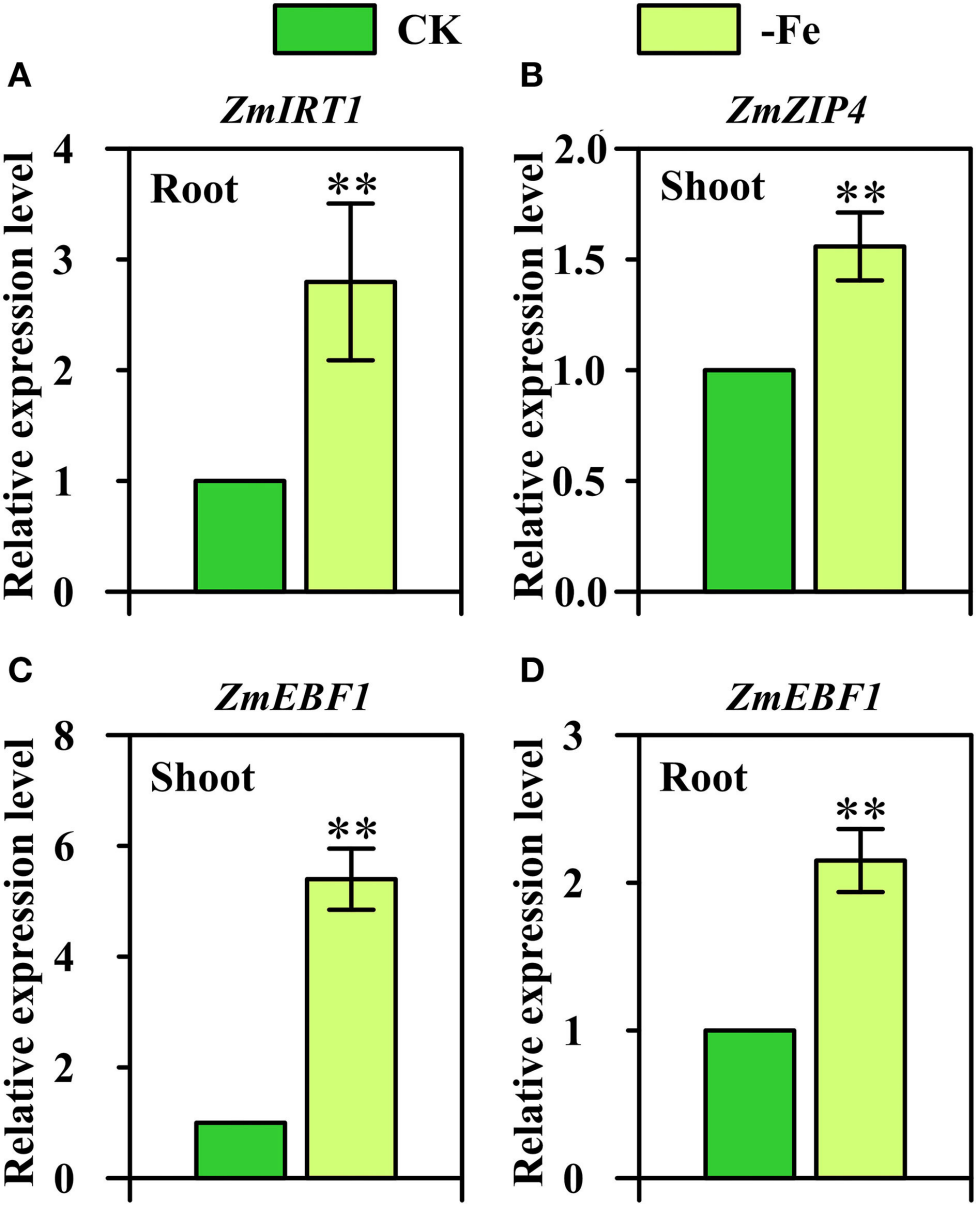
Relative expression levels of strategy I genes **(A)** *ZmIRT1* (GRMZM2G118821), **(B)** *ZmZIP4* (GRMZM2G111300), and **(C,D)** *ZmEBF1* (GRMZM2G171616) in the shoots **(B,C)** and/or roots **(A,D)** of Ye478 grown under Fe-deficient (-Fe) and Fe-sufficient (CK) conditions. ** Indicates significant differences between treatments at *P* < 0.01.

Interestingly, three strategy I genes displayed Fe-deficiency-inducible upregulation in maize which is considered as the strategy II plant (Figure 7). *ZmIRT1* (GRMZM2G118821) was found to be significantly upregulated in the roots under Fe deficiency (Figure 7A). Furthermore, the expression of *ZmZIP4* (GRMZM2G111300) displayed a 1.5-fold upregulation in the shoots in response to low Fe stress (Figure 7B). *ZmEBF1* (GRMZM2G171616), which encodes EIN3-binding F-Box protein 1 and plays a key role in the ethylene signaling pathway and Fe homeostasis of strategy I plants, was found to be significantly upregulated in both shoots and roots under Fe deficiency (Figures 7C,D).

## Discussion

### Comparisons of Loci Identified in This Study With Previous Reports

To date, numerous studies concentrate on the concentrations of Fe and other minerals in different tissues of maize (Qin et al., 2012; Šimić et al., 2012; Zdunić et al., 2014; Gu et al., 2015; Zhang et al., 2017; Hindu et al., 2018; Ma et al., 2021). However, there were some reports of mapping the QTLs associated with shoot and root biomass accumulation, yield, plant height, and root traits (Azevedo et al., 2015; Burton et al., 2015; Li P. et al., 2016; Wang et al., 2016; Luo et al., 2017). Therefore, the loci identified in these previous studies were compared with the current study based on the physical position of each significant SNP.

In total, 113 of 353 significant SNPs identified by GWAS have been found to be co-localized with the reported QTLs or SNPs in previous studies (Supplementary Table S4). Among these loci, five significant SNPs which were associated with root dry weight and R/S ratio were co-localized with two significant SNPs associated with leaf necrosis under Fe-deficient condition detected by GWAS (Benke et al., 2015). In addition, eight QTLs related to root length, shoot and root dry weight, and R/S ratio detected in the current study were overlapped by previously reported QTLs or SNPs associated with iron (Fe), zinc (Zn), and magnesium (Mn) concentration in grains detected by linkage analysis or GWAS (Gu et al., 2015; Hindu et al., 2018), suggesting that these co-localized genomic regions may have pleiotropic effects on Fe and other mineral concentration of grains in maize.

In addition, a total of 53 significant SNPs associated with Fe score, plant height, root length, shoot and root dry weight, and R/S ratio detected in the present study was co-localized with the loci controlling plant height under salt stress or normal conditions reported by Luo et al. (2017). Besides, 64 significant SNPs controlling six different traits in this work overlapped with ten loci controlling root morphology under low-phosphorus (P) stress detected by Azevedo et al. (2015) and nitrogen (N) stress identified by Li P. et al. (2016). These findings indicate that these genomic regions may harbor several genes with pleiotropic effect on plant height or root morphology under abiotic stress at the maize seedling stage.

Moreover, the Fe-deficiency-inducible candidate gene *ZmNAAT1*, containing the R/S-associated SNP, was co-localized with the QTL controlling ear height, leaf angle, and yield in maize, and the candidate gene *ZmDMAS1*, which was markedly upregulated by Fe deficiency in shoots and roots (Figure 7B), was identified within the co-localization of the significant chr1.S_31743584 associated with root dry weight identified in this work and the QTL controlling root diameter under low-phosphorus stress reported by Azevedo et al. (2015). These results further confirm that these genomic regions may be highly valuable for identifying the genes associated with Fe-deficiency tolerance.

### Candidate Genes Maintain Fe Homeostasis in Strategy II Plants

Strategy II plants acquire Fe through mugineic acid family phytosiderophores (MAs) (Bashir and Nishizawa, 2006). In the MA biosynthetic pathway, nicotianamine aminotransferase (NAAT) and deoxymugineic acid synthase (DMAS) enzymes catalyze the formation of 2'-deoxymugineic acid (DMA) from nicotianamine (NA) (Takahashi et al., 2003), and then, DMAs are secreted into the rhizosphere (Li et al., 2019). Subsequently, Fe(III)-DMA and Fe(II)-NA complexes are taken up from the rhizosphere by transporters, such as Yellow Strip 1 and Yellow Stripe1-Like proteins (Curie et al., 2001; Inoue et al., 2009). In this study, strategy II genes were identified, including *ZmNAAT1, ZmDMAS1*, and *ZmYSL11*.

*OsNAAT1*, a homolog of *ZmNAAT1*, was upregulated in all cells of Fe-deficient roots and shoots, and its transcripts were more abundant in yellow young leaves than in green old leaves under Fe deficiency (Cheng et al., 2007; Inoue et al., 2009). Herein, we further confirm that *ZmNAAT1* expression was not only induced by Fe-deficiency stress in roots, but also in shoots (Figures 6A,B). DMAs have been detected in xylem and phloem, and the amount of DMA is reported to increase in Fe-deficient shoots and roots (Nishiyama et al., 2012; Ariga et al., 2014; Nozoye et al., 2014). *ZmDMAS1, OsDMAS1, HvDMAS1*, and *TaDMAS1* are highly homologous to each other and are all strongly upregulated under Fe-deficient condition (Bashir and Nishizawa, 2006; Benke et al., 2014; Beasley et al., 2017). Furthermore, *OsDMAS1* expression is restricted to the cells participating in long-distance transport under Fe-sufficient condition and is detected in vascular bundles specifically under Fe-deficient condition (Bashir et al., 2006). More importantly, our results further confirm that *ZmDMAS1* responded to Fe deficiency more in shoots than in roots (Figures 6C,D), which was inconsistent with the results of gene expression analysis performed by Nozoye et al. (2013).

*YSL* genes, such as *AtYSL1* and *OsYSL2*, are reported to transport metals via NA complexes, including Zn(II)-NA, Cu(II)-NA and Fe(II)-NA, Mn(II)-NA (DiDonato et al., 2004; Koike et al., 2004; Le Jean et al., 2005; Ishimaru et al., 2010). *OsYSL15* is found to encode an Fe-specific transporter which is not only responsible for Fe uptake from the rhizosphere but also for phloem transport of Fe by Fe(III)-DMA (Inoue et al., 2009; Lee et al., 2009). In this study, *ZmYSL11* was furthermore confirmed to be significantly upregulated by Fe deficiency in both shoots and roots (Figures 6E,F).

### Strategy I Genes May Be Involved in Fe-Deficiency Tolerance in Maize

In this study, genes involved in strategy I, including *ZmIRT1, ZmZIP4*, and *ZmEBF1*, were identified within the genomic regions flanking the significant SNPs detected by GWAS (Table 1). There is basically no research on these genes in maize. However, the function summary of the homologous genes of these genes in other plants is helpful to understand their functions in maize. Beyond that, we also analyzed their expression pattern in response to Fe deficiency in maize.

IRT1 is the bifunctional transporter-receptor at the heart of metal sensing and signaling, and IRT1 transcription in response to low iron is dually controlled by local and long-distance signals (Cointry and Vert, 2019). In this research, we further confirm that *ZmIRT1* was Fe-deficiency-inducible in the roots of maize inbred line (Figure 7A).

The exact role of most *ZIP* members in planta has not been understood, including *ZmZIP4*. *OsZIP4* and, its close homolog, *OsZIP3*, are both highly expressed in the nodes. OsZIP3 is mainly involved in unloading Zn from the xylem of enlarged vascular bundles (Sasaki et al., 2015). OsZIP4 is involved in transporting Zn to the phloem of diffuse vascular bundles in the nodes for subsequent distribution to the developing tissues, including tiller buds, new leaf, and panicles (Mu et al., 2021). It also functions in transporting Zn to meristem cells in the tiller buds. Our expression results showed that *ZmZIP4* was significantly upregulated in the Fe-deficient roots of maize (Figure 7B).

So far, the role of hormones and signaling substances on the regulation of Fe-deficiency responses in strategy II species has been rarely studied (Lucena et al., 2015). Under Fe deficiency, ethylene is implicated in the activation of some Fe-related genes (Kobayashi et al., 2014). The responses of strategy I plants to ethylene are mediated by regulation of EBF1/2-dependent degradation of the ETHYLENE INSENSITIVE3 (EIN3) transcription factor, which is critical for growth in plants and has been analyzed by many studies (Gagne et al., 2004; An et al., 2010). Moreover, in the current study, we found that *ZmEBF1* was not only significantly upregulated in the shoots, but also in the roots (Figures 7C,D). These findings suggest that strategy I genes, namely *ZmIRT1, ZmZIP4*, and *ZmEBF1*, may be involved in the mechanisms underlying Fe-deficiency tolerance in maize.

## Data Availability Statement

The original contributions presented in the study are included in the article/Supplementary Material, further inquiries can be directed to the corresponding author/s.

## Author Contributions

JX and WX performed the experiments and wrote the manuscript. JX, WX, XC, and XF analyzed data. FY conceived the project and designed the research. All authors contributed to the article and approved the submitted version.

## Funding

This study was supported by the National Key Research and Development Program of China (2016YFD0200405).

## Conflict of Interest

The authors declare that the research was conducted in the absence of any commercial or financial relationships that could be construed as a potential conflict of interest.

## Publisher's Note

All claims expressed in this article are solely those of the authors and do not necessarily represent those of their affiliated organizations, or those of the publisher, the editors and the reviewers. Any product that may be evaluated in this article, or claim that may be made by its manufacturer, is not guaranteed or endorsed by the publisher.
