# Supplementary material for: Genome-Wide Association Analysis Reveals the Genetic Basis of Iron-Deficiency Stress Tolerance in Maize
Source: Front Plant Sci. 2022 Jun 2;13:878809. doi: 10.3389/fpls.2022.878809 (PMC9202619; doi:10.3389/fpls.2022.878809)
Supplement: Supplementary file 1 [file Table_1.docx]

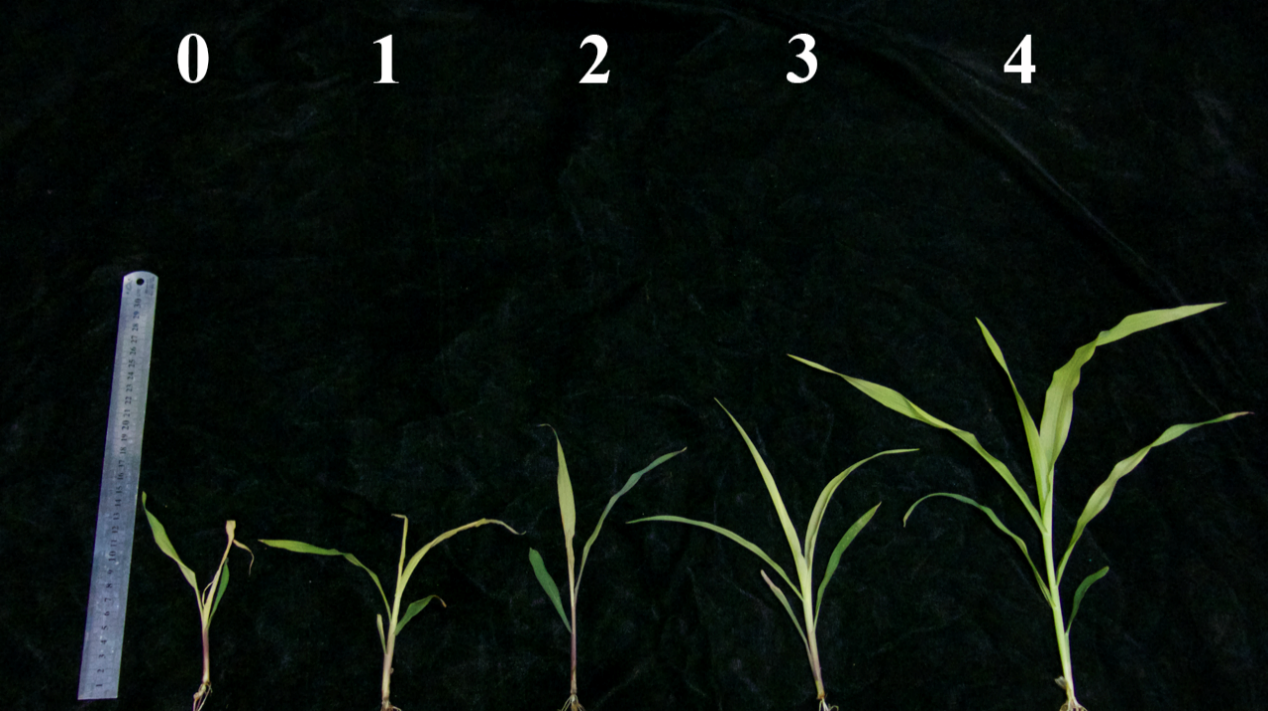
**Figure S1** Fe score for each plant under Fe deficiency has been visually recorded for three times since 12th day after transplanting. Five scales (0 - 4) are designed to assess the Fe deficiency tolerance of maize at seedling stage. Score 0: plants developed about three leaves with one sprout, and showed the severest stunted growth and reduced plant height, distorted leaf growth as well as yellowish-white necrotic lesions distributed on the whole youngest leaves. Score 1: plants developed four leaves and one sprout, and showed reduced plant height and wrinkled leaf margins. Fe-deficient chlorosis with necrotic patches was distributed on the youngest leaves. Score 2: plants developed four leaves and one sprout, and exhibited a better growth compared with Score-0 and Score-1 plants, but still showed chlorosis on youngest leaves. Score 3: plants developed four leaves and one sprout, and obtained an improved plant height compared with Score-2 plants. Young leaves showed Fe-deficient chlorosis without wrinkled leaf margins. Score 4: plants grew well without reductions in plant height but still display banded chlorosis on the middle of young leaves.


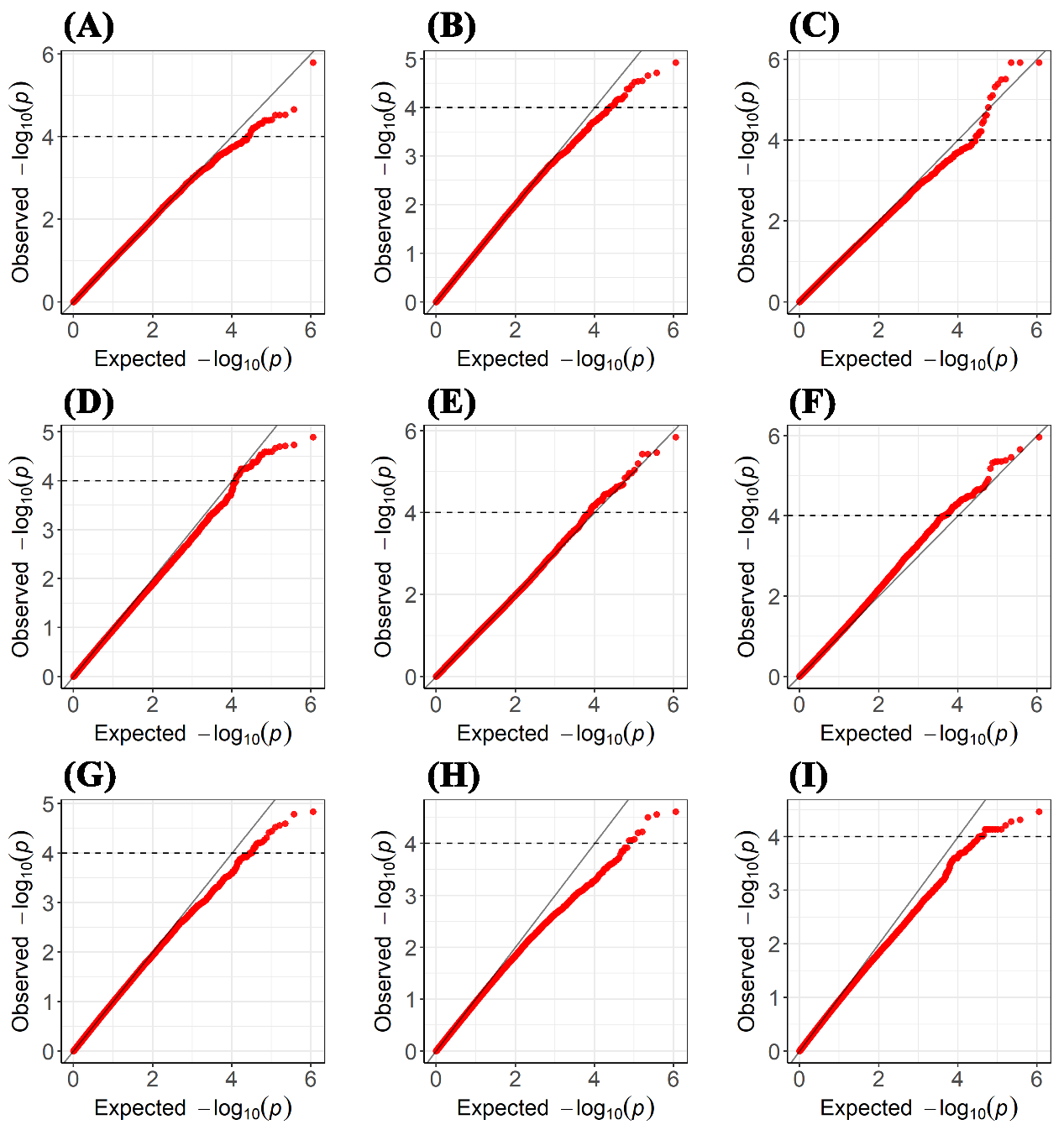


**Figure S2** Quantile-Quantile plots for **(A)** Fe score, **(B)** plant height, **(C)** root length, (-Fe: **(D)**; -Fe/CK: **(G)**) shoot dry weight, (-Fe: **(E)**; -Fe/CK: **(H)**) root dry weight, (-Fe: **(F)**; -Fe/CK: **(I)**) R/S ratio.
